# Enhanced Water Resistance of TiO_2_–GO–SMS-Modified Soil Composite for Use as a Repair Material in Earthen Sites

**DOI:** 10.3390/ma17184610

**Published:** 2024-09-20

**Authors:** Wei Li, Wenbo Bao, Zhiqiang Huang, Yike Li, Yuxuan Guo, Ming Wang

**Affiliations:** 1 School of Materials Science and Engineering, Shenyang University of Technology, Shenyang 110870, China; 2 School of Architecture and Civil Engineering, Shenyang University of Technology, Shenyang 110870, China; 3 School of Materials Science and Engineering, Liaoning Technical University, Fuxin 123000, China

**Keywords:** nano-TiO_2_/GO/SMS multi-component modification, water resistance performance, mechanical properties, micro-modified mechanism

## Abstract

Most earthen sites are located in open environments eroded by wind and rain, resulting in spalling and cracking caused by shrinkage due to constant water absorption and loss. Together, these issues seriously affect the stability of such sites. Gypsum–lime-modified soil offers relatively strong mechanical properties but poor water resistance. If such soil becomes damp or immersed in water, its strength is significantly reduced, making it unviable for use as a material in the preparation of earthen sites. In this study, we achieved the composite addition of a certain amount of sodium methyl silicate (SMS), titanium dioxide (TiO_2_), and graphene oxide (GO) into gypsum–lime-modified soil and analyzed the microstructural evolution of the composite-modified soil using characterization methods such as XRD, SEM, and EDS. A comparative study was conducted on changes in the mechanical properties of the composite-modified soil and original soil before and after immersion using water erosion, unconfined compression (UCS), and unconsolidated undrained (UU) triaxial compression tests. These analyses revealed the micro-mechanisms for improving the waterproof performance of the composite-modified soil. The results showed that the addition of SMS, TiO_2_, and GO did not change the crystal structure or composition of the original soil. In addition, TiO_2_ and GO were evenly distributed between the modified soil particles, playing a positive role in filling and stabilizing the structure of the modified soil. After being immersed in water for one hour, the original soil experienced structural instability leading to collapse. While the water absorption rate of the composite-modified soil was only 0.84%, its unconfined compressive strength was 4.88 MPa (the strength retention rate before and after immersion was as high as 93.1%), and the shear strength was 614 kPa (the strength retention rate before and after immersion was as high as 96.7%).

## 1. Introduction

Earthen sites, which are a special category of historical and cultural heritage, are composed mainly of earthen materials. These sites, together with their surroundings, record the historical information of the era in which they are situated, retain the imprints of their epochal characteristics, and are an important reference for the retrospective restoration of the area’s original historical features. Given the scientific, historical, artistic, and non-renewable nature of earthen sites, they must be preserved to the greatest extent possible. The earthen sites in Liaoning Province are primarily composed of powdery clay, with uneven particle grading and a substantial number of interconnected pores. These characteristics confer certain strength properties in a dry state but are susceptible to damage such as pulverization and disintegration when exposed to water, freeze–thaw cycles, and other environmental factors [1]. The traditional raw soil material exhibits low strength, poor water resistance, poor volume stability, and other properties that significantly limit its application. Wan [2], Hou [3], and other researchers have identified these limitations. The incorporation of desulfurized gypsum, fly ash, and hydrated lime within raw soil materials significantly enhances compressive strength and water resistance. After 30 min of water absorption saturation, the compressive strength of these modified materials can reach one-third of their original strength. Sodium methyl silicate, titanium dioxide, and graphene oxide can also be added to enhance the waterproof and mechanical properties of the material. This method represents a highly effective protection measure that can be maximized to protect earthen sites or raw earth buildings.

Sodium methyl silicate (SMS) was shown to effectively enhance the hydraulic properties of various building materials [4,5,6,7]. For example, Ma [8] analyzed natural hydraulic limestone by adding potassium methyl silicate and organic silicon. When the potassium methyl silicate content was 0.3%, the natural hydraulic lime soil offered good mechanical properties and durability, indicating the optimal ratio. He [9] investigated the influence of SMS content on the water resistance of desulfurized gypsum. The authors found that adding 1% SMS had minimal impact on the gypsum’s strength but significantly reduced its water absorption rate from 20.63% (in the control group) to 7% and increased its softening coefficient from 0.72 (in the control group) to 0.85, indicating a marked improvement in water resistance. Cao [10] studied silt from a cemetery site in Luoyang and found that adding 2% nano-silica and 0.5% SMS significantly enhanced both the mechanical and water-resistant properties of the material. Lv [11] explored the hydrophobic modification effects of composite SMS on the gel skeleton of alkali slag, finding that hydrophobic groups (-CH_3_) could be grafted onto the chemical structure of C–A–S–H/C–S–H gel through condensation reactions. Overall, most studies have applied SMS to modern building materials such as concrete, ceramics, and gypsum, while research on the use of SMS in modifying earthen building materials remains limited.

Research has explored enhancements to the mechanical and durability properties of cement-based materials through the addition of titanium dioxide (TiO_2_) [12,13,14,15,16]. Babaei [17] discovered that a clay mixture optimized with 1.48% TiO_2_, 6.04% cement, and 20% kaolinite exhibited cohesion of 215.3 kPa, a friction angle of 49.78°, and an unconfined compressive strength of 1.503 MPa. Anuj [18] investigated the strength and durability of clay treated with a combination of nano-silica and polypropylene fibers, noting that the uniaxial compressive strength grew as the quantity of these additives increased. Duan [19] demonstrated the successful application of nano-TiO_2_ in protecting the surfaces of historical stone relics. This research showed that the high specific surface area of nano-TiO_2_ particles could adsorb harmful substances surrounding the relics and that its potent photocatalytic degradation capability could swiftly break down these harmful substances.

Graphene oxide (GO), an innovative nanomaterial and the oxidized form of graphene, exhibits a sheet-like structure. Recently, the application of this material in modifying cement and clay mixtures has gained substantial attention [19,20,21,22,23,24]. Tong [25] investigated the enhancement mechanisms of GO via microstructural characterization, analyzing the microstructure of the cement paste surrounding GO nanoparticles and constructing an atomic model of GO-reinforced C–S–H gel. These findings demonstrated that GO can significantly enhance freezing resistance. Ammar [26] demonstrated that using GO as an alleviating agent has a significant impact on the physical features of expansive soil, making this material beneficial in the construction of roads, dams, and bridges. Aziz [27] studied the effects of graphene oxide nanomaterials on mechanical properties such as the compaction characteristics, elastic modulus, UCS, and microstructural characteristics of a soil–cement composite. Gao [28] reported that incorporating GO into clay cement paste enhanced its properties by promoting nucleation, accelerating the cement hydration reaction, and generating more C-S-H within microcapillary pores, while also bridging microcracks in the material.

The present study utilizes sodium methyl silicate (SMS), TiO_2_, and GO as additives to modify S0 (Original soil). Through unconfined compressive strength and triaxial compression tests, we explore how SMS, TiO_2_, and GO influence the compressive strength, shear strength, and strength retention of S0 materials after water erosion. This research analyzes the water resistance of S0 modified with SMS, TiO_2_, and GO, shedding light on the mechanisms underlying its water resistance and ability to enhance mechanical performance.

## 2. Experimental Section

### 2.1. Specimen Preparation

In this study, the clay near Qiansuo wall in the western Liaoning Province of northeast China is used as the research object.

Original soil S0 is a mixture of clay mixed with 15% desulfurized gypsum, 5% fly ash, 5% hydrated lime, and 0.5% jute fiber. The compressive strength and shear properties of this mixture are significantly better than those of raw soil materials [3].

Sodium methyl silicate is a colorless transparent liquid with a solid content (%) of ≥30 and a pH value of 12–13. This liquid is an organic compound with good permeability and crystallinity.

The nano TiO_2_ used in the test was produced by Liaoning Quanrui Reagent Co., LTD as a crystal form of anatite, with a purity of 99.8%, a controlled size of 40 nm, a melting point of 1840 °C, a boiling point of 2900 °C, a density of 4.26 g/mL (25 °C environment), and a white powdery appearance. GO solution was prepared using the improved Hummers’ method [29].

All specimens in this study were molded using a *φ*39.1 mm × H80 mm cylindrical mold under a pressure of 30 KN, compacted in three stages. After compacting each layer, the surface was planned to ensure even contact between layers before proceeding to the next stage of compaction. The moisture content of the specimen during preparation was controlled within ±0.02%. The materials were air-dried and subsequently oven-dried to achieve a moisture content of 4.8% for testing.

### 2.2. Experimental Method

(1) Water immersion resistance test

This experiment investigated the water resistance enhancement effects of SMS, TiO_2_, and GO on the multi-component composite material S0. For this analysis, we examined the water absorption characteristics of samples over a one-hour period. Samples that disintegrated within one hour were deemed to have poor water resistance, while those that remained intact were considered to have good water resistance. The relative water absorption rate *R* (%/min) is calculated as $R=\frac{M_{T+10}-M_{T}}{M_{T}*10}\times 100\%$. Where *M_T_* is the mass of the sample after immersion for *T* min, *M* is the quality of the sample after curing for 28 days, and *T* is the soaking time. Water absorption Q (%) is calculated as $Q=\frac{M_{60}-M}{M}\times 100\%$. Where *M*_60_ is the mass of the sample after soaking for 60 min, and *M* is the quality of the sample after curing for 28 days.

(2) Unconfined Compressive Strength Test

Using a conventional stress–strain controlled triaxial testing apparatus, we assessed the compressive strength of natural and modified soils. The tests were conducted at a loading rate of 1 mm/min and completed within 10 min. Sample dimensions were *φ*39.1 mm × H80 mm. Procedures adhered to the guidelines specified in the “Standard Test Methods for Soil Mechanics” (GB/T50123-2019) [30], yielding a stress–strain curve for the unconfined compressive strength of the soil.

The uncollapsed sample was placed in a natural environment for 28 days, after which its strength change was measured to calculate the strength retention rate. The strength retention rate *K* (%) is calculated as $K=\frac{I_{60}}{I_{0}}\times 100\%$. Where *I*_60_ is the unconfined compressive strength of the sample after 60 min of immersion in water, MPa; and *I*_0_ is the unconfined compressive strength (MPa) of the specimen after 28 days of curing.

(3) Triaxial shear test

A TSZ-1 strain-controlled triaxial testing machine from Nanjing Soil Instrument Factory was utilized to perform unconsolidated undrained (UU) triaxial shear tests on 28-day-old samples; the same test was adopted from Eddine [31]. The shear rate during testing was set to 0.5 mm/min, and each test in the same series involved adjusting only the confining pressure. Testing was concluded upon reaching an axial strain of 15%.

After placing unconsolidated undrained (UU) samples in natural conditions for 28 days, changes in the cohesion and internal friction angle for different modified materials were analyzed using the shear strength envelope curve. The retention rates of the cohesion and internal friction angle were calculated accordingly.

The calculation formula for the cohesion retention rate *K*_1_ (%) is $K_{1}=\frac{c_{60}}{c_{0}}\times 100\%$. Where *C*_60_ is the cohesion of the sample after 60 min of immersion in water, MPa; and *C*_0_ is the cohesion of the specimen after curing for 28 days (MPa). The variation of the internal friction angle was obtained by comparing the tangents of the internal friction angle for different modified soils.

(4) XRD characterization

The phase analysis of the samples in this study was performed using an X-ray diffractometer (XRD, XRD-6100, Kyoto, Japan), with a Cu target producing *Kα* radiation at *λ* = 0.154178 nm. The instrument operated at 40 kV and 30 mA, scanning over a 2*θ* range from 5° to 90° at a rate of 10° min^−1^. The Braggs equation 2*d*sin*θ* = *nλ* was applied, where λ denotes the wavelength of the X-ray, *θ* is the diffraction angle, *d* represents the interplanar spacing, and n indicates the diffraction order.

(5) SEM analysis

Surface morphology observations of the samples in this study were performed using a scanning electron microscope (SEM, Phenom ProX, Eindhoven, Holland). The samples were first dispersed in anhydrous ethanol via ultrasound and then fixed on copper pillars using a conductive adhesive after the anhydrous ethanol had evaporated, enabling morphological observations.

(6) EDS analysis

The EDS analysis was carried out on an X-ray spectrometer (EDS, EDAX-9100, Erfurt, Germany) manufactured by SEM (supra55 FESEM, Erfurt, Germany). The test standard was based on “Technical Specifications for Protection Test of Earth Sites” [32].

## 3. Results and Discussion

### 3.1. Microstructure Characterization

**Figure 1:**
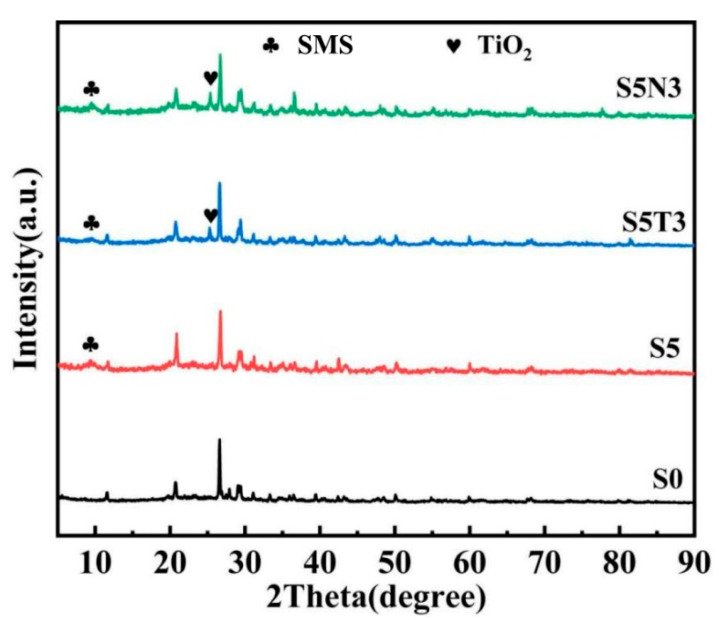
XRD patterns of different modified soils.

The crystal structures of the samples were investigated using X-ray diffraction (XRD), with the resulting XRD patterns illustrated in Figure 1. Analysis of the figure reveals the lack of a peak around 10° in the XRD pattern of S0, indicating the absence of SMS. Conversely, weak diffraction peaks near 10° are evident in the XRD patterns of S5, S5T3, and S5N3, suggesting the presence of SMS. In the XRD pattern of S5N3, a prominent peak appears at approximately 10°, corresponding to the (002) crystal plane characteristic of graphene oxide (GO). Other diffraction peaks also closely resemble those of S5. A distinct weak peak at 25.5° can also be observed in the XRD pattern of S5T3, attributed to the (101) crystal plane of well-crystallized rutile-type TiO_2_, indicating successful loading onto S5 with high crystallinity. Diffraction peaks of both GO and TiO_2_ are visible in the XRD pattern of S5N3, suggesting that the introduction of GO does not hinder the formation of rutile-phase TiO_2_. The intensity of TiO_2_ diffraction peaks in S5N3_3_ is higher than that in S5, albeit with a slight shift towards lower angles at 25.5°, possibly indicating intercalation of TiO_2_ and GO within the interlayers of S5.

**Figure 2:**
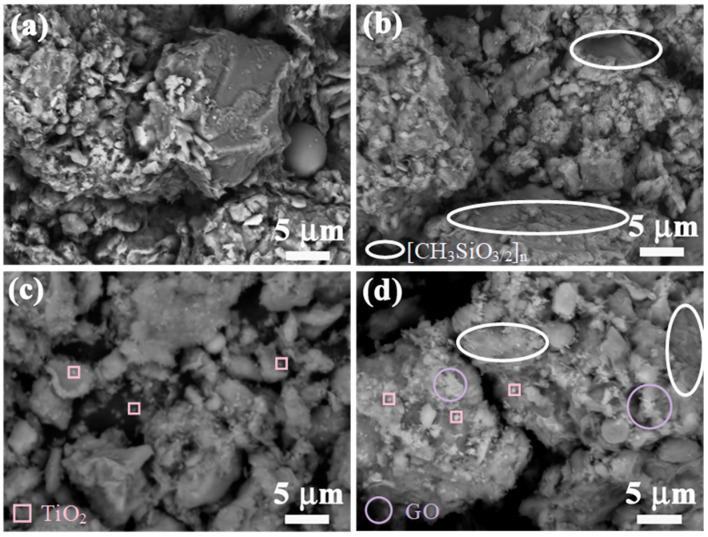
SEM images of different modified soils: (**a**) S0; (**b**) S5; (**c**) S5T3; (**d**) S5N3.

Figure 2a,b shows the surface morphology of S0 and S5. In Figure 2b, the ${\left[{\mathrm{CH}}_{3}{\mathrm{SiO}}_{\frac{3}{2}}\right]}_{n}$ membrane is circled with a white ellipse. SMS, gypsum, lime, fly ash, and undergo a series of physical and chemical reactions in original soil, that improving its mechanical properties and water resistance.

SMS reacts with water and CO_2_ to form a $\left({\left[{\mathrm{CH}}_{3}{\mathrm{SiO}}_{\frac{3}{2}}\right]}_{n}\right)$ film. This film has strong hydrophobicity and can increase the contact angle on the surface of restoration soil particles, in addition to improving their water resistance. The corresponding chemical reaction formulae are as follows:

(1) $$2{\mathrm{CH}}_{3}\mathrm{Si}{\left(\mathrm{OH}\right)}_{2}\mathrm{ONa}+{\mathrm{CO}}_{2}+H_{2}O\to 2{\mathrm{CH}}_{3}\mathrm{Si}{\left(\mathrm{OH}\right)}_{3}+{\mathrm{Na}}_{2}{\mathrm{CO}}_{3}$$

(2) $${\mathrm{nCH}}_{3}\mathrm{Si}{\left(\mathrm{OH}\right)}_{3}\to {\left[{\mathrm{CH}}_{3}{\mathrm{SiO}}_{\frac{3}{2}}\right]}_{n}+\frac{3}{2H_{2}O}$$

These membranes are interconnected near the soil particles to form a larger, more complete membrane structure, which strengthens the inter-particle connections, reduces pore space, and blocks the pathway for external water infiltration. This hydrophobic organosiloxane membrane also significantly enhances the water resistance of the soil while maintaining good air permeability, thereby conforming to the principles of soil site remediation.

SMS has the largest effect on the index of water resistance. Compared with the original soil, with an increase in SMS doping, the cohesion and the internal friction angle of the modified soil tends to decline, while the reduction rate of the water-absorbing mass presents a gradual upward trend. The methyl group in each molecule of Sodium methyl silicate is a common hydrophobic group that can form a water-repellent layer on the surface of restorative soil particles. This layer can facilitate micro-expansion and increase compactness.

Figure 2c shows an image of S5T3, in which TiO_2_ is framed by pink squares. Due to the gradual increase in TiO_2_ content, many small particles appeared between the ${\left[{\mathrm{CH}}_{3}{\mathrm{SiO}}_{\frac{3}{2}}\right]}_{n}$ membranes, which appear white under an electron microscope. The presence of TiO_2_ also improves the catalytic performance of the catalyst. Nano titanium dioxide can effectively absorb ultraviolet rays and prevent them from penetrating the protective layer, thereby protecting earthen sites artifacts. Moreover, nano titanium dioxide has superhydrophilic interfacial properties; as its content increases, the hydrophobicity and permeability of the protective layer also increase, thus enhancing the layer’s “breathability”. As noted by Giovanni, this property facilitates the preservation of ceramics and stone artifacts [33].

Figure 2d shows an image of S5N3, in which GO is marked by a purple circle. Nanocomposite modification via the addition of SMS greatly increased the strength of S5N3 compared to the results when using composite modification alone. The presence of GO contributed to the formation of rough wrinkled laminar structures, suggesting that compositing with S5 increased the spacing of the layers and provided active sites. In addition, the presence of TiO_2_ resulted in a large number of small, uniformly distributed particles on the surface of S5, which improved the catalytic performance of the catalyst.

**Figure 3:**
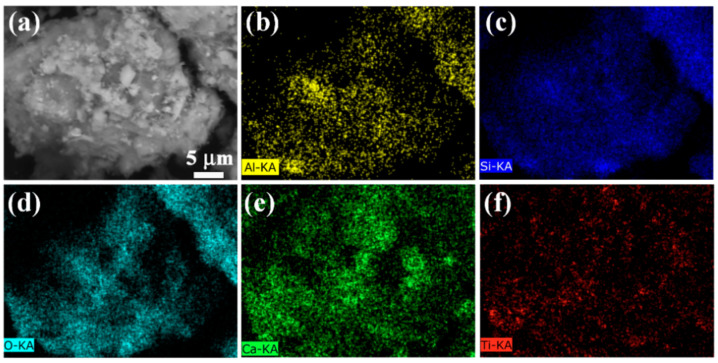
EDS mapping scanning images of S5N3: (**a**) surface morphology; (**b**) Al; (**c**) Si; (**d**) O; (**e**) Ca; (**f**) Ti.

SEM combined with EDS enables a more comprehensive analysis of the elemental composition and distribution in S5N3 nanocomposite materials. Figure 3 presents the EDS spectra of the S5N3 nanocomposite materials, illustrating the uniform distribution of Al, Si, Ca, O, and Ti elements across the material. This uniformity suggests the formation of a cohesive network structure among TiO_2_, GO, and S5. Table 1 illustrates, in detail, the elemental composition and contents of the composite material. Here, the percentages of O, Ca Si, Al, and Ti are 21.51%, 53.00%, 10.06%, 10.05%, and 5.38%, respectively. This result aligns with the SEM analysis results in showing that the composite material is S5N3.

### 3.2. Water Immersion Resistance Performance

**Figure 4:**
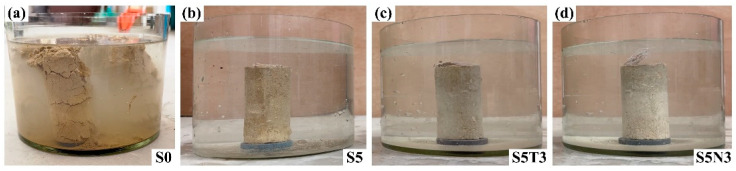
The macroscopic appearance of different modified soil specimens soaked in water for 1 h: (**a**) S0; (**b**) S5; (**c**) S5T3; (**d**) S5N3.

As shown in Figure 4, S0 contains clay blended with desulfurized gypsum, fly ash, hydrated lime, and hemp knife specimens. When submerged in water, the material did not immediately disintegrate due to the protective effects of ettringite and hydrated calcium sulfate products formed via the hydration of fly ash and lime, which encapsulated the gypsum crystal framework. Figure 4a illustrates the water immersion test for S0, in which disintegration appeared at the top after 30 min, indicating that the water resistance performance still requires improvement.

In recent years, sodium methyl silicate has been studied for its ability to improve the water-resistance of soil sites [34,35]. To enhance the hydraulic performance of S0, different amounts of sodium methyl silicate were added. Then, we investigated the effects of these additions on the water resistance of the modified soil. Due to its hydrophobic nature, the specimen’s surface produced large air bubbles that repel water, causing some areas of the specimen to remain isolated from water contact. As shown in Figure 4b, after 50 min of immersion, S5 exhibited small bubbles, and, after one hour, partial surface soil detachment occurred, with no large bubbles observed throughout the test. Figure 4c,d shows that S5T3 and S5N3 presented minimal bubble formation after 50 min of immersion and no surface soil detachment after one hour, with no occurrence of large bubbles throughout the entire duration of the test.

**Figure 5:**
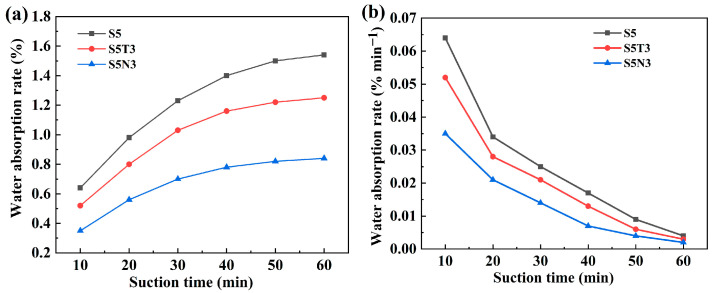
(**a**) Variation curve of water resistance for 1 h of different modified soil; (**b**) variation curve of water absorption rate of different modified soil.

Figure 5 presents the variable curve for water absorption rate of different modified soil. Sodium methyl silicate significantly enhanced the water resistance of S0, reducing the water absorption rate of S5 to 1.54% after one hour of immersion. Incorporating TiO_2_ and GO maintained the structural stability of S0, resulting, respectively, in an 18.8% and 45.5% reduction in the water absorption rate for S5T3 and S5N3 over 60 min compared to the results for S5. As immersion time increased, the relative water absorption rate of S_5_ gradually decreased, saturating after 40 min. S5T3 reached saturation after 30 min, while S5N3 achieved saturation after 20 min. Sodium methyl silicate contributed the most to enhancing water resistance, primarily due to the methyl groups in its molecules, which are common hydrophobic groups. These groups can form hydrophobic layers on the surface of modified soil S0 particles, providing functions such as slight expansion and increased compactness [8]. Incorporating TiO_2_ and GO maintained S5’s excellent structural stability while reducing its relative water absorption rate.

### 3.3. Mechanical Properties

**Figure 6:**
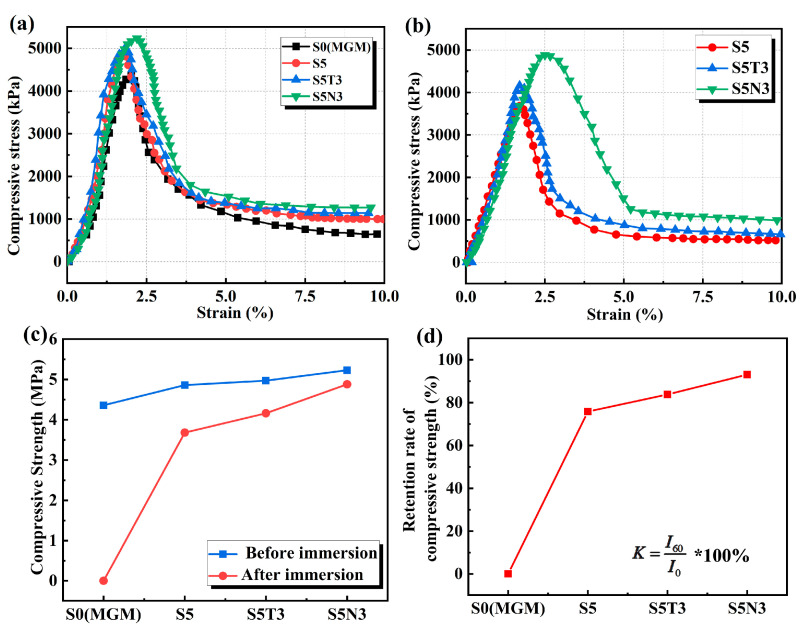
Compressive stress–strain curve of different modified soils (**a**) before immersion and (**b**) after immersion; (**c**) comparison of the compressive strength of different modified soils before and after immersion; (**d**) retention rate of the compressive strength for different modified soils immersed in water for one hour.

Unconfined compressive strength (UCS) serves as a key indicator for evaluating the modification and stabilization effects of treated soils. Conducting UCS tests enabled us to obtain the stress–strain curves of four types of treated soil materials before and after immersion in water, as presented in Figure 6a,b, which illustrates the stress–strain behavior under UCS tests for the four materials regarding unconfined compressive strength. The stress–strain curves of S0, S5, S5T3, and S5N3 under axial load clearly present softening characteristics. Stress initially increases with strain, reaches a peak, and subsequently decreases gradually, converging towards residual strength. There was no significant change in the stress–strain curve characteristics before and after immersion. The peak strain of the S0 stress–strain curve decreased from 2% to 1.7%, while the peak strains of S5 and S5T3 remained largely unchanged. For S5N3, the peak strain increased from 2.2% to 2.5%. This result suggests that the addition of nanomaterials has a minimal impact on the peak unconfined compressive strength of materials after immersion but enhances their resistance to deformation, similar to the results of Foad et al. [36]. Nanomaterials can mitigate the failure and cracking of modified soil [37].

As seen in Figure 6c,d, before immersion, the UCS of S5 was 11.5% higher than that of S0 after 28 days. The addition of sodium methyl silicate significantly enhanced S0. Lu found that the hardness and strength of geopolymers increased after the addition of SMS [38]. The UCS of S5T3 presented a minor increase, while S5N3, after the addition of GO, showed a 19.95% increase in compressive strength compared to that of S0. This result occurred because nanomaterials filled the gaps between S0 particles, thereby increasing internal density and enhancing the compressive strength of the modified soil. Similar observations were reported by Li et al. [39]. After one hour of immersion, S0 disintegrated, while the strength of the other three materials decreased due to water absorption and expansion. After curing for 28 days, the retention rates of unconfined compressive strength (UCS) compared to pre-immersion rates were 75.8% for S5, 83.8% for S5T3, and 93.07% for S5N3. S5N3 provided the best retention rates for UCS, from 5.23 MPa to 4.88 MPa.

**Figure 7:**
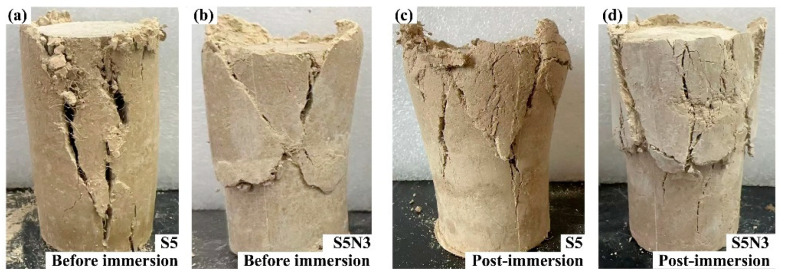
Triaxial shear failure macroscopic appearance of modified soil: (**a**) S5 before immersion; (**b**) S5N3 before immersion; (**c**) S5 post-immersion; (**d**) S5N3 post-immersion.

Unconsolidated undrained (UU) triaxial tests were conducted to determine the shear strength and stress–strain relationships of different modified soils. As shown in Figure 7, S5 presented a combination of localized swelling and localized shear failure, both before and after immersion. Post-immersion, the failure zones exhibited noticeable deformation, indicating that one hour of immersion had a detrimental effect on the structure of S5, with the material structure remaining somewhat loosened after curing. S5N3 exhibited X shear failure before immersion and a combination of swelling and multiple shear failures after immersion, suggesting minimal structural damage to S5N3 after one hour of immersion.

**Figure 8:**
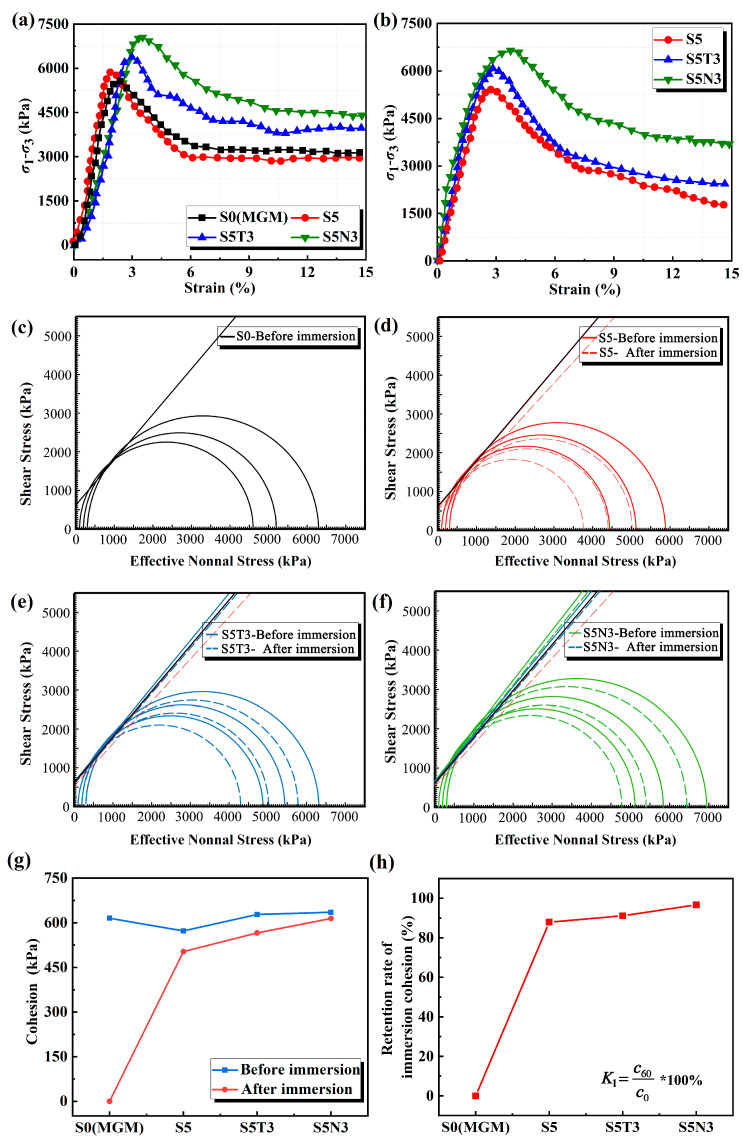
Triaxial stress–strain curves of different modified soils at 300 Kpa (**a**) before immersion; (**b**) after immersion; (**c**) Mohr circles for S0; (**d**) Mohr circles for S5; (**e**) Mohr circles for S5T3; (**f**) Mohr circles for S5N3; (**g**) comparison of the cohesive stress of different modified soils before and after immersion; (**h**) retention rate of the cohesive stress of different modified soils immersed in water for one hour.

Figure 8a,b shows the triaxial stress–strain curves of different modified soils at 300 kPa. Figure 8a shows that, before immersion, as the strain increased, the stress of S0 and S5 increased rapidly to the peak stress and then decreased rapidly and tended to remain stable. The failure mode was a strain-softening type, indicating that the material possessed a certain fragility. As the strain increased, the stress of S5T3 and S5N3 increased rapidly to the peak stress and then decreased relatively gently. The peak strain for S5N3 was 3.2%, and for S0 was 2.2%. With the addition of nanomaterials, the shear resistance of S5N3 increased, and the displacement curve of the failure points moved to the right [40]. Figure 8b shows the post-immersion results. S0 disintegrated; the stress of S5, S5T3, and S5N3 increased rapidly with an increase in strain and then decreased rapidly after the peak strain; the peak strain was between 3 and 3.5%; and the peak stress was universally lower than that before immersion.

Figure 8c–h shows a shear strength envelope diagram of the cohesive strength and internal friction angle derived for S0, S5, S5T3, and S5N3, respectively. Figure 8c–f clearly shows that the cohesive and internal friction angle of S5, S5T3, and S5N3 decreased after immersion. Before immersion, the cohesive strength of S5 was equal to 572.7 kPa and it decreased by 7% compared to that of S0. The addition of SMS reduced the material’s shear strength, with similar observations reported by Ma et al. [8]. The cohesive strength of S5T3 and S5N3 increased by 2% and 3.3%, respectively, compared to that of S0. The tangents of the internal friction angle were almost constant between S0 and S5, and an increase in friction angle was observed after adding nano-TiO_2_ and GO. Figure 8g,h shows the post-immersion results. The cohesive strength of S5 decreased by 12.15% compared to the pre-immersion value, while S5T3 decreased by 10% and S5N3 decreased by 3.34%. The change in the internal friction angle for the S5N3 tangents before and after immersion was less than that of S5, with similar expressions reported by Driss et al. [31]. The combined use of TiO_2_ and GO notably enhanced the shear strength of S5 after immersion better than that before immersion, with a retention rate increase of nearly 9%. S5N3 offered the best retention rates for cohesion, which ranged from 635.61 kPa to 614 kPa [41].

After modifying S0 with 5% SMS, the SMS solution reacted with water and carbon dioxide, decomposing into methyl silicate which rapidly formed a poly (methyl siloxane) film covering the surfaces of the soil particles. This film enhanced the waterproofing performance of the modified soil. S5 presented excellent mechanical properties and water resistance. Following water erosion and a 28-day rest period, the cohesive strength of this soil showed a slight decrease, indicating that the water resistance and mechanical properties of S0 were significantly improved by adding nano-TiO_2_/GO and SMS. Similar results on the modification of silty soil sites using nanosilica and methylsilicate were reported by Cao et al. [10]. S5N3 exhibited minimal changes in shear strength before and after immersion, thereby maintaining soil stability.

### 3.4. Structural Evolution Mechanism of S5N3

**Figure 9:**
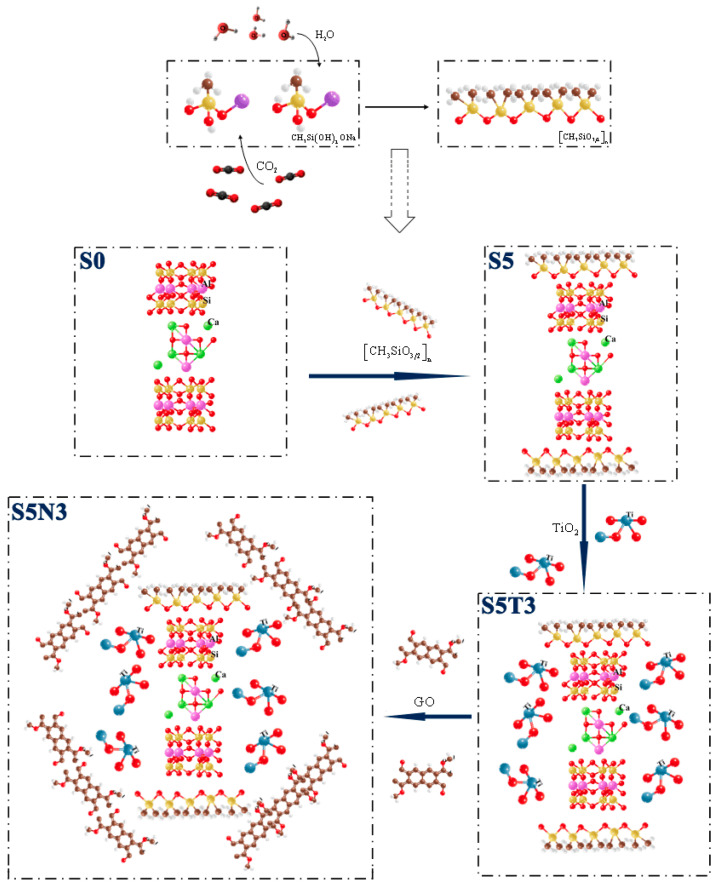
The structural evolution mechanism schematic of S5N3.

The structural evolution mechanism schematic for S5N3 is shown in Figure 9.

#### 3.4.1. The Formation Mechanism of S0 (Original Soil)

We divided the hydration process using desulfurized gypsum, fly ash, and hydrated lime as modified materials into two stages as follows:

The first stage involved desulfurized gypsum hydration. After mixing with water, desulfurized gypsum rapidly combined with water to generate gypsum dihydrate crystals, which constituted the skeleton of the whole system and formed strength early in the process [42]. The fly ash hydration rate was very slow, so fly ash hydration was ignored at this stage. In addition, fly ash was used only as an aggregate to fill the skeleton gap. Ultimately, the desulfurized gypsum–fly ash–hydrated lime system offered high strength at an early stage.

The second stage involved fly ash hydration. This process was relatively slow, as fly ash was gradually activated and hydrated in the already-hardened slurry. The hydration mechanism was as follows. Since the desulfurized gypsum formed the crystal skeleton of gypsum dihydrate, the whole slurry had poor fluidity, a low concentration of Ca^2+^ and OH^−^, and a relatively slow hydration rate. Moreover, the products generated by fly ash hydration, calcium alumina, and hydrated calcium sulfate were only used to fill voids in the dihydrate gypsum skeleton.

The role of hydrated lime in the desulfurized gypsum–fly ash–hydrated lime-modified material is to provide Ca^2+^ and OH^−^. The limited hydration of fly ash in this system yielded a reduction in the OH^−^ requirement. At the same time, it was necessary to ensure that the admixture of fly ash remained at a relatively high level.

It is generally believed that the process of sulfate excitation in the fly ash and hydrated lime system can be divided into three stages. The first stage involves the surface contact reaction. Under the joint action of Ca(OH)_2_ and sulfate, the active SiO_2_ on the surface of the fly ash particles produces a hydration reaction to generate I-type CSH gel. The active Al_2_O_3_ then produces a hydration reaction that generates calcium alumina, some hydration, CSH gel, and calcium alumina deposited on the surface of the fly ash particles, which form an encapsulated layer. This layer impedes the absorption of calcium ions by the fly ash particles, slowing the hydration rate. In the second stage, Ca^2+^ absorbs energy for diffusion penetration, the inner molecules absorb energy (“energy storage”), and the surface of the CSH and calcium alumina crystal experiences growth. Due to the hindering effect of the parcel layer, there is a relative decrease in the reaction rate. This slowdown is the key step affecting the speed and degree of fly ash activity excitation and is mainly affected by three factors: First, this process relies on the reaction environment temperature. Although the solubility of Ca(OH)_2_ reduces with an increase in temperature, temperature changes at room temperature do not have a large impact on the solubility of Ca(OH)_2_. However, an increase in temperature provides a large amount of energy, enabling the parcel layer inside and outside the ions store energy. Here, temperature is high, and Ca^2+^ achieves high energy levels and diffusion speed, resulting in a very rapid slowdown period. Second, this process is impacted by the structure of the surface parcel layer of fly ash particles. The more perfect the fibrous and reticulate structure of the surface parcel layer is, the more rapidly the smoothing period ends. The third factor relates to the nature of the fly ash itself. Under the same external conditions, the more active the components of the fly ash are, the shorter the plateau period will be. The third stage involves osmotic pressure, ionic “energy storage”, and other factors. At this stage, the parcel layer ruptures, and fly ash active ingredients continue to hydrate to generate CSH and calcium alumina crystals. At this stage, the system continues to grow and improve, and the strength of the slurry continues to increase. The same description was proposed by Ahmed M. Abdelbaset [43].

#### 3.4.2. S5 Reaction Mechanism

SMS, desulfurized gypsum, fly ash and lime in the soil produce a series of physical and chemical reactions and gradually form a membrane above and below S0, as shown in Figure 9. These processes change the nature of the soil, thereby improving its mechanical properties and water resistance.

SMS has the smallest effect on the index of cohesion and internal friction angle of the repair soil and the largest effect on the index of water resistance compared with that of original soil. With an increase in the dosage of sodium methyl silicate, the cohesion and the internal friction angle of the modified soil tends to decline, while the rate of reduction in the water-absorbing mass presents a gradual increasing trend. The methyl group in the molecule of sodium methyl silicate is a common hydrophobic group that can form a water-repellent layer on the surface of restorative soil particles and facilitates micro-expansion in addition to increasing compactness.

Sodium methyl silicate reacts with water and CO_2_ to form a polysiloxane film, which has strong hydrophobicity and can increase the contact angle on the surface of the repair soil particles as well as improve their water resistance [38]. The relevant chemical reaction formulae are provided in Equations (1) and (2).

#### 3.4.3. S5T3 Evolution Mechanism

With a gradual increase in TiO_2_ content, many small particles appeared to fill ${\left[{\mathrm{CH}}_{3}{\mathrm{SiO}}_{\frac{3}{2}}\right]}_{n}$. Through this process, hydration filled the soil body [44], and the soil particles became connected to form a dense S5T3 structure, as shown in Figure 9, effectively improving the tightness and integrity of the S5T3 and greatly improving the material’s waterproofing performance.

#### 3.4.4. S5N3 Evolution Mechanism

The addition of GO filled the internal pores of S5T3 at the nanoscale and prevented the further development of cracks, forming a dense S5N3 membrane inserted at the voids, as shown in Figure 9. GO has nanoscale filling and bridging anti-cracking effects, which can prevent cracks and voids during the bonding process of hydration products and soil particles from expanding from the nanoscale to the micrometer scale in order to improve the denseness between S5T3 [45]. Additionally, GO’s highly active oxygen-containing functional groups can promote a hydration reaction, optimize cement hydration, and generate an abundant C–S–H gel structure, thereby improving the strength and volume stability of the raw soil material. When GO/TiO_2_/SMS materials combine together, hydration fills the soil and connects the soil particles to form a dense structure that has difficulty absorbing water, leading to structural damage.

#### 3.4.5. Synergistic Hydration Effect

For S5N3, hydration products such as ettringite (AFt) and calcium hydroxide (CH), which are insoluble in water, serve as the foundation for nucleation reactions with GO as the core. The large specific surface area and high reactivity of GO allow it to adsorb incompletely reacted desulfurized gypsum, fly ash, lime particles, and original soil particles during hydration, thereby becoming the focal point of hydration reactions. This feature promotes the formation of orderly and dense hydration reaction products, enhancing overall structural integrity. When GO and TiO_2_ are co-blended, they synergistically promote and leverage their advantages, which helps enhance the water resistance and mechanical properties of S0.

#### 3.4.6. Ion-Exchange Adsorption Effects

The ionic exchange effect is another significant mechanism underlying the synergistic action of S5N3. The double electric layer structures on the surfaces of clay particles contain abundant K^+^ and Na^+^. During the hydration process of the S0 mixture, the produced Ca^2+^ exchanged with K^+^ and Na^+^ on the soil particle surfaces, reducing the quantity of K^+^ and Na^+^ ions on the raw soil surface. Furthermore, the introduction of higher valence ions like Ca^2+^ decreased the thickness of the raw soil’s double electric layer, thereby enhancing the binding force between raw soil particles and improving the microstructure and mechanical properties of the raw soil-based materials. Moreover, GO facilitated the S0 hydration process, producing a significant amount of hydration products and Ca^2+^ ions under similar conditions. This strengthened the chemical modification and ion exchange effects between the raw soil particles, further increasing the bonding strength between them. This increased strength ensured a more robust and dense structure and enhanced the material’s mechanical properties.

#### 3.4.7. Filling and Water Resistance Effect

For S0 (15% desulfurized gypsum + 5% fly ash + 5% hydratedlime + 0.8% jute fiber), the large amounts of hydration products generated during hydration fill the voids between raw soil particles and connect them to form a structural framework with certain strength [46]. This framework prevents the formation and development of internal cracks within the raw soil particles, effectively improving the compactness and integrity of the raw soil materials [47]. [CH_3_SiO_3/2_]_n_/TiO_2_/GO exhibits nano-filling and bridging anti-cracking effects, preventing the expansion of intruding water from the nanoscale to the microscale in the process of bonding hydration products with soil particles, thereby enhancing the compactness between the hydration products and raw soil materials. When [CH_3_SiO_3/2_]_n_/TiO_2_/GO are co-blended, hydration fills the soil mass and connects the soil particles to form a dense structure. TiO_2_-GO then fills the internal pores of the raw soil materials at the nanoscale, preventing further water development. The synergistic effect of this process enhances the erosion resistance and mechanical properties of S5N3.

## 4. Conclusions

In this paper, SMS, TiO_2,_ and GO were added to an original soil. Then, the composite-modified soil was studied via waterproofing performance and mechanical property tests. The modification mechanisms of SMS, TiO_2,_ and GO were investigated from a microscopic perspective through SEM, EDS, and XRD testing, providing a theoretical basis for the protection and restoration of earthen sites in the Liaoning area. The relevant conclusions of this study are as follows:

(1) Compared with S0 (original soil), the water resistance performance was effectively enhanced after the addition of SMS, TiO_2_, and GO. The water absorption rate of S5N3 was only 0.84% (S0 collapsed structurally after 30 min of immersion). This result was attributed to $\left({\left[{\mathrm{CH}}_{3}{\mathrm{SiO}}_{\frac{3}{2}}\right]}_{n}\right)$ film formation from SMS, which developed a water-repellent layer on the surface of the restorative soil particles and improved their water resistance.
(2) The addition of SMS, TiO_2_, and GO effectively enhanced the mechanical properties of S0 (original soil), in which S5N3 retained 93.07% of its post-immersion unconfined compressive strength and 96.54% of its post-immersion cohesion after 28 days of curing. This result was dominated by coupled synergistic hydration, nano-filling, and bridging anti-cracking effects from the [CH_3_SiO_3/2_]_n_/TiO_2_/GO nano-composited component.

## Figures and Tables

**Table 1:** EDS diagram comparison table of S5N3 composite materials.

Element	Atomic Percent
O	55.63
Si	26.11
C_a_	4.71
Al	11.85
Ti	1.70
Weight	100.00

## Data Availability

Data is contained within the article.
